# Systematic Review: Are Table-Top Roleplaying Games a Useful Therapeutic Tool for People with Neurodiversity?

**DOI:** 10.1192/bjo.2025.10240

**Published:** 2025-06-20

**Authors:** Madison Thompson, Vivek Majumder

**Affiliations:** Cardiff University, Cardiff, United Kingdom

## Abstract

**Aims:** This study aimed to review prior research on tabletop role-playing games (TTRPG) as a therapeutic tool for neurodiverse individuals and identify gaps in the field to direct future research.

**Methods:** A comprehensive systematic review of available literature on the use of TTRPGs in the neurodiverse population was completed, with no limitations to publication language or date. This was completed using appropriate medical subject headings (MeSH terms) and ProQuest to search 21 electronic data bases. Papers were superficially reviewed using the title and abstract to determine if they met the inclusion criteria and relevant articles were reviewed in full.

**Results:** The systematic search resulted in the identification of 5 relevant articles, containing a combination of peer-reviewed journal articles (n=3), and dissertations/theses (n=2). The majority of papers (n=3) were published in the western world, with others coming from Japan (n=1) and Brazil (n = 1).

**Conclusion:** Existing literature suggests that TTRPGs offer significant benefits for neurodiverse individuals, particularly in enhancing social skills, communication, and overall social functioning. Kato’s (2019) qualitative case study showed that TTRPGs improved social functioning, intentional communication, and cooperative decision-making in youth with autism spectrum disorder (ASD). Henning et al. (2024) echoed these findings, though they were not statistically significant. The benefits of TTRPGs were also demonstrated by Parks (2021), remarking that TTRPGs allow autistic young adults to practice social roles and skills at their own pace. Atherton et al. (2024) expanded this by showing that TTRPGs helped autistic adults form social connections in a safe, structured setting, promoting community and friendship. A shift in focus was seen in the study presented by Rubin-Budick (2022), which focused on the perspective of the therapist over the participants. This research gave an overriding consensus on the effectiveness of TTRPGs in improving emotional attunement and social engagement. Whilst the current body of research highlights these potential benefits, it is important to note that the studies conducted so far have been limited by small sample sizes and narrow participant demographics, with an overriding focus on ASD over other neurodiverse conditions.

Overall, available literature suggests that TTRPGs can enhance social development, communication, and emotional well-being in neurodiverse individuals. This review has also highlighted the need to explore the role of TTRPGs as a therapeutic tool in a wider range of neurodiverse conditions, with particular reference to attention deficit hyperactivity disorder (ADHD).

